# Efficacy of SMTP‐7, a small‐molecule anti‐inflammatory thrombolytic, in embolic stroke in monkeys

**DOI:** 10.1002/prp2.448

**Published:** 2018-12-05

**Authors:** Eriko Suzuki, Naoko Nishimura, Tetsuya Yoshikawa, Yudai Kunikiyo, Keiko Hasegawa, Keiji Hasumi

**Affiliations:** ^1^ Department of Applied Biological Science Tokyo Noko University (Tokyo University of Agriculture and Technology) Tokyo Japan; ^2^ Division of Research and Development TMS Co., Ltd. Tokyo Japan; ^3^ Shin Nippon Biomedical Laboratories, Ltd. Kagoshima Japan

**Keywords:** cerebral infarction, embolic stroke, inflammation, SMTP‐7, t‐PA

## Abstract

SMTP‐7 (*Stachybotrys microspora* triprenyl phenol‐7) is a small molecule that promotes thrombolysis and suppresses inflammation possibly through plasminogen modulation and soluble epoxide hydrolase (sEH) inhibition, respectively. Here, we demonstrate an efficacy of SMTP‐7 in a severe embolic stroke model in monkeys. The middle cerebral artery was embolized by an autologous blood clot. Saline, SMTP‐7, or tissue‐type plasminogen activator (t‐PA) (n = 5 in each group) was given after 3 hours, and neurologic deficit scoring and infarct characterization were performed after 24 hours. Hemorrhagic infarct‐accompanied premature death was observed for two animals in t‐PA group. SMTP‐7 treatment significantly reduced the sizes of infarct by 65%, edema by 37%, and clot by 55% compared to saline treatment. Plasma levels of the products of plasminogen activation (plasmin‐α_2_‐antiplasmin complex) and sEH reaction (dihydroxyeicosatrienoic acid) in SMTP‐7 group were 794% (*P* < 0.05) and 60% (*P* = 0.085) compared to saline group, respectively. No significant changes in the plasma levels of MMP‐9, CRP, MCP‐1, and S100B were found. There was an inverse correlation between plasmin‐α_2_‐antiplasmin complex level and infarct volume (*r* = 0.93, *P* < 0.05), suggesting a role of thrombolysis in the SMTP‐7 action to limit infarct development. In conclusion, SMTP‐7 is effective in treating severe embolic stroke in monkeys under conditions where t‐PA treatment tends to cause hemorrhagic infarct‐associated premature death.

**Abbreviation**
ARRIVE: Animal Research: Reporting In Vivo Experiments
AU: arbitrary unit
CRP: C‐reactive protein
DHET: dihydroxyeicosatrienoic acid
IQR: interquartile range
MCA: middle cerebral artery
MCP‐1: monocyte chemoattractant protein‐1
MMP‐9: matrix metalloproteinase‐9
PAP: plasmin‐α_2_‐antiplasmin complex
S100B: protein S100‐B
sEH: soluble epoxide hydrolase
SMTP‐7: *Stachybotrys microspora* triprenyl phenol‐7
t‐PA: tissue‐type plasminogen activator
TTC: 2,3,5‐triphenyltetrazolium chloride

## 1 INTRODUCTION

Thrombolytic therapy is a powerful means to treat ischemic stroke.^1^ Currently, the standard method is the use of the enzyme tissue‐type plasminogen activator (t‐PA), which specifically cleaves plasminogen, converting it to the active thrombolytic enzyme plasmin.^2^, ^3^ There are, however, some limitations in the use of t‐PA, because it occasionally leads to bleeding complications including fatal intracranial hemorrhage.^4^ The major limitation is the time window of its use: within 3 to 4.5 hour following symptom onset, depending on regional regulation or guideline.^4^ Good outcome declines, and bad outcome increases along with the time to the t‐PA treatment, and the 3‐ to 4.5‐hour time window is the critical lines to benefit patients who receive t‐PA.^4^ The major cause of the time‐dependent drawback is postulated to be ischemia‐reperfusion injury, which leads to neuronal cell death, cerebral inflammation, and eventually blood‐brain barrier disruption.^5^

SMTP, named after *Stachybotrys microspora* triprenyl phenol, is a family of small molecules derived from the fungus *S. microspora*.^6^ SMTP was first discovered by a screening of microbial cultures for a compound that promoted plasminogen binding to fibrin, a crucial step of physiological fibrinolysis.^6^ SMTP‐7 (2,5‐bis[2‐[(3*E*)‐4,8‐dimethylnona‐3,7‐dienyl]‐3,5‐dihydroxy‐2‐methyl‐7‐oxo‐4,9‐dihydro‐3*H*‐pyrano[2,3‐*e*]isoindol‐8‐yl]pentanoic acid), one of the most intensively studied SMTP congeners, relaxes plasminogen conformation and, thereby, enhances plasminogen‐fibrin binding and plasminogen activator‐catalyzed activation of plasminogen,^7^, ^8^, ^9^ leading to a promotion of thrombus clearance in vivo.^8^, ^10^ On the other hand, SMTP‐7 exhibits anti‐inflammatory activities in vivo in disease models that are irrelevant to thromboembolism.^11^ Inhibition of soluble epoxide hydrolase (sEH) may account for the anti‐inflammatory mechanism.^11^, ^12^

SMTP‐7 is effective in treating several types of ischemic stroke models in rodents. These include thrombotic and embolic strokes as well as transient or permanent mechanical ischemia of the brain.^13^, ^14^, ^15^, ^16^, ^17^, ^18^ The efficacy of SMTP‐7 in the mechanical ischemia models suggests a significant role of its anti‐inflammatory action. Furthermore, experiments using several analogs of SMTP suggest that anti‐inflammatory and anti‐oxidative properties, as well as the profibrinolytic action, contribute to the therapeutic activity of SMTP.^19^, ^20^ The risk of SMTP‐7 to compromise hemostasis is far low compared to that of t‐PA.^17^ The difference in the mechanism of action of the two agents may account for this result. SMTP‐7 enhances endogenous fibrinolytic process through modulating plasminogen conformation,^6^ whereas t‐PA directly activates plasminogen. Although t‐PA exacerbated ischemia‐reperfusion‐associated cerebral hemorrhage, SMTP‐7 ameliorated cerebral hemorrhage in a mouse model where animals were pretreated with the anticoagulant warfarin.^16^ In addition, time window of SMTP‐7 treatment is broader than that of t‐PA treatment as observed in embolic stroke models in rodents.^14^, ^18^

Stroke is heterogeneous both in its cause and clinical course.^21^ Therefore, animal testing using various models in higher order species that resemble clinical setting is beneficial to predict clinical efficacy of a drug candidate.^22^, ^23^ Regarding thrombotic stroke, we observed an efficacy of SMTP‐7 in a photochemical thrombosis model in cynomolgus monkeys.^17^ Along with thrombosis, another major cause of ischemic stroke is thrombotic embolization.^24^ We, therefore, attempted to test SMTP‐7 in an embolic stroke model in monkeys to confirm its efficacy. The timing of drug treatment was set at 3 h after thrombus embolization where t‐PA failed to yield good outcome. We also explored clinically relevant biomarkers that predict outcome based on the mechanism of action, such as plasminogen activation and inhibition of sEH.

## 2 MATERIALS AND METHODS

### 2.1 Drugs

SMTP‐7 was produced by *S. microspora* 25 and converted to sodium salt (lot N01YHN, purity 99.19%). Recombinant t‐PA (ACTIVACIN for Injection) was obtained from Kyowa Hakko Kirin, Tokyo, Japan. SMTP‐7 (10 mg/kg), t‐PA (3 mg/kg), and vehicle (saline) were administered intravenously (5 mL/kg) as follows: bolus injection of 10% of the total dose for 5 seconds followed by infusion of the remaining dose over 30 minutes (for SMTP‐7 or saline) or bolus injection of 10% of the total dose for 1 minute followed by infusion of the remaining dose over 60 minutes (for t‐PA). The decision to set the doses of SMTP‐7 and t‐PA were based on the results from preliminary experiments using the cynomolgus monkey embolic stroke model. The t‐PA dose setting was supported by the fact that its fibrinogenolytic activity in monkeys at 3 mg/kg (reducing plasma fibrinogen level to 60% of control)^26^ was comparable to that in humans at a clinically relevant dose (60 mg/body; reducing plasma fibrinogen level to 57% of control).^27^

### 2.2 Animal experiment

The animal study was performed in compliance with the Standards for the Reliability of Application Data (Article 43, Enforcement Regulations, Pharmaceutical Affairs Law, March 23, 2005, the Ministry of Health, Labour and Welfare, Japan, Ordinance No. 37). This study was approved by the Institutional Animal Care and Use Committee (approval No. IACUC345‐016) and was performed in accordance with the animal welfare bylaws of the Drug Safety Research Laboratories of Shin Nippon Biomedical Laboratories, Ltd. (Kagoshima, Japan), which is fully accredited by the Association for Assessment and Accreditation of Laboratory Animal Care International. All the animal protocols complied with the ARRIVE (Animal Research: Reporting In Vivo Experiments) guidelines. We took adequate steps to ensure that animals did not suffer unnecessarily at any stage of the experiment.

Male cynomolgus monkeys obtained from Angkor Primates Center Inc. (Par Sat, Cambodia) and Tian Hu Cambodia Animal Breeding Research Center Ltd. (Sdoeung Chey, Cambodia) were bred at Shin Nippon Biomedical Laboratories, Ltd. The monkeys were housed in cages that were 68‐cm‐wide by 62‐cm‐high by 77‐cm‐deep (conforming to the NIH requirements) and kept under a 12‐hour light/dark cycle. Animals (4‐6 years old, 3.53‐5.54 kg) were acclimated for 4 days.

Animals (n = 28) underwent a catheter implantation in the left internal carotid artery for clot injection under the successive treatments with atropine sulfate (0.02 mL/kg, i.m.), ketamine hydrochloride (10 mg/kg, i.m.), and then buprenorphine hydrochloride (10 g/kg, i.m.), followed by inhalation of isoflurane (0.5%‐2.0%). The catheter (0.86 mm in internal diameter) was filled with heparin (500 IU/mL) and tunneled subcutaneously into the neck toward the dorsal region. Animals received an antibiotic mixture once daily (12.5 mg/kg dihydrostreptomycin sulfate and 10 000 U/kg benzylpenicillin procaine) and buprenorphine hydrochloride (10 g/kg) twice daily for 3 days. On the third day, blood (3 mL) was drawn from the femoral vein and incubated in a catheter (1.67 mm in internal diameter) at 37°C for 3 hours to allow clotting. The autologous clot formed (approximately 10 cm in length) was flushed through the catheter placed in the left internal carotid artery with 0.6 mL of saline to embolize the middle cerebral artery (MCA) under the anesthesia with propofol (1%, 0.4 mL/kg, i.v.) and isoflurane inhalation (0.5‐2.0%). We set the following criteria for exclusion: (1) any animal judged unsuitable as a model 3 hours after embolization based on the neurologic deficit score 28 (Table S1) and clinical sign observed 1 and 3 hours after the embolization; and (2) any animal judged unsuitable to continue the study during the observation period (until 24 hours after embolization). Details of the exclusion criteria are shown in Table 1. We excluded four ineligible animals based on the exclusion criterion 1. The results of the exclusion are summarized in Table 1.

**Table 1:** Exclusion criteria and cases in animal testing

Exclusion criteria		Excluded animal
(1) Any animal judged unsuitable as a model 3 h after embolization based on:		Total: n = 4
(i)	neurologic deficit score of 6 or below for consciousness or skeletal muscle coordination, or clinical sign symptomatic of anterior cerebral artery occlusion (unable to maintain sitting position due to right lower limb extension)	#2, #27
(ii)	neurologic deficit score of 16 or greater in the consciousness category	
(iii)	neurologic deficit score of 16 or greater in the skeletal muscle coordination category	#1, #13
(iv)	total neurologic deficit score of 47 or greater, and any two of the following abnormalities: vomitus, severe salivation, and muscle weakness in ipsilateral limb (left upper and lower limbs)	
(v)	exclusion is judged necessary by the study director	
(2) Any animal judged unsuitable to continue the study during the observation period (after dosing until 24 h after embolization) based on:		Total: n = 9 Saline group: n = 3 SMTP‐7 group: n = 3 t‐PA group: n = 3
(i)	neurologic deficit score of 16 or greater in the consciousness category	
(ii)	neurologic deficit score of 16 or greater in the skeletal muscle coordination category	Saline group: #12 t‐PA group: #4
(iii)	total neurologic deficit score of 47 or greater, and any two of the following abnormalities: vomitus, severe salivation, and muscle weakness in ipsilateral limb (left upper and lower limbs)	
(iv)	exclusion is judged necessary by the study director	
(v)	premature death in saline group	#9, #14
(vi)	premature death without intracranial hemorrhage in SMTP‐7 group or t‐PA group	SMTP‐7 group: #7, #10, #28 t‐PA group^a^: #6, #24

There was no statistical significance in the differences in the number of excluded animals among the 3 groups when analyzed by χ^2^ test. The animal number corresponds to the picture number in Figure S1.

Any animal to which one of the following criteria conforms were to be excluded from the study. Twenty‐eight animals (number #1 to #28, ascending order from the lowest body weight) were subjected to embolization. After excluding 4 animals (#1, #2, #13, and #27), 24 animals were randomized to saline (#3, #9, #12, #14, #17, #19, #21, and #23), SMTP‐7 (#5, #7, #10, #15, #20, #22, #25, and #28), and t‐PA (#4, #6, #8, #11, #16, #18, #24, and #26) groups. For humane reason, the excluded animals were euthanized by an intravenous injection of sodium pentobarbital (64.8 mg/mL, 0.4 mL/kg), followed by exsanguination.

a There were two more prematurely died animals in the t‐PA group (#8 and #18). These had massive hemorrhagic infarct and were regarded as “dosing‐related death,” which were not excluded from the study.

The eligible animals (n = 24) were randomly assigned to three groups (n = 8 for each group). The drug dosing was made after 3 hours of the thrombus embolization. The treatment time point was set based on our preliminary experiments and the knowledge from the literature that showed irreversible brain damages after 2‐3 hours of severe ischemia in a monkey transient ischemia model.^29^ We scored neurologic deficits at 5, 7, 18, and 24 hours following the embolization. The observer was not aware of any information on individual animals including treatment assignment. Nine animals that met the exclusion criterion 2 during the observation period were excluded from the study (Table 1). Macroscopic appearance of the origin of the MCA in the excluded animals is shown in Figure S1. Premature death with hemorrhagic infarct in t‐PA or SMTP‐7 groups was not to be excluded but to be counted as a “dosing‐related death.” This settlement was due to the fact that t‐PA treatment caused hemorrhagic infarct‐associated premature death in some animals in the previous experiments conducted at the Shin Nippon Biomedical Laboratories using this embolic stroke model. In this study, there were two cases that met the dosing‐related death criterion in the t‐PA group (Table 1).

The survived animals (n = 13) were anesthetized by an intravenous injection of sodium pentobarbital (26 mg/kg) and perfused with cold saline from the left ventricle to draw blood 24 hours after the thrombus embolization. Subsequently, the brain was removed, and its external appearance including the origin of the MCA was photographed, followed by preparation of successive 14 coronal brain slices (4‐mm thickness). The sections were inspected macroscopically for hemorrhage and stained with 1% 2,3,5‐triphenyltetrazolium chloride (TTC). Digital images of the stained sections were recorded to quantitate infarct area using an image analysis software (analySIS, Soft Imaging System, Münster, Germany). Infarct area and the left hemispheric area in each slice was determined, and the sum of the data for the 14 slices, after multiplying by a factor of 4, were regarded as infarct volume and expressed as a percentage of the ipsilateral hemispheric volume. The degree of edema (%) was calculated as: (volume of ipsilateral hemisphere – volume of contralateral hemisphere)/volume of contralateral hemisphere × 100. The size of clots remaining in the MCA region was evaluated by analyzing photographs using ImageJ after normalizing background. The product of the normalized color depth over area was regarded as the mass of the thrombus, which was expressed in arbitrary unit (AU). All the evaluations were performed in a blinded manner.

### 2.3 Bleeding time

Bleeding time was measured twice, immediately after the thrombus embolization and immediately after the end of dosing under isoflurane anesthesia (0.5%‐2.0%). Part of the antebrachial region was shaved and incised using a bleeding device (ITC Surgicutt™ Bleeding Time Device, Fisher Scientific, Waltham, MA). A filter paper was applied to the site of incision every 30 seconds to determine the time until cessation of bleeding. Two successive observations of the absence of bleeding were regarded as bleeding cessation, and the time for the first observation was taken as the bleeding time.

### 2.4 Blood parameters

The plasma levels of the products of plasminogen activation (plasmin‐α_2_‐antiplasmin complex; PAP) and sEH (dihydroxyeicosatrienoic acid; DHET), as well as substances related to inflammation, including matrix metalloproteinase‐9 (MMP‐9), C‐reactive protein (CRP), monocyte chemoattractant protein‐1 (MCP‐1), and protein S100‐B (S100B), were determined using blood drawn just before the completion of the drug infusion (for PAP and DHET) or blood drawn 24 hours after the thrombus embolization (for MMP‐9, CRP, MCP‐1, and S100B). PAP was determined using a zymographic method as described previously.^17^ Four regioisomers of DHET [(±)‐5,6‐DHET, (±)‐8,9‐DHET, (±)‐11,12‐DHET, and (±)‐14,15‐DHET] were determined using an LC‐MS/MS method.^11^ MMP‐9, CRP, MCP‐1, and S100B were determined by enzyme‐linked immunosorbent assays using commercially available kits (Human MMP‐9 Quantikine ELISA kit, R&D Systems, Minneapolis, MN; Human C‐Reactive Protein ELISA kit, Merck Millipore, Tokyo, Japan; Human MCP‐1/CCL2 ELISA MAX™ Deluxe kit, Bio Legend, San Diego, CA; and S100B Human ELISA kit, BioVendor, Brno, Czech Republic, respectively).

### 2.5 Data analysis

Data for neurologic deficits scores and bleeding time are indicated as the median and the interquartile range (IQR). Infarct size, and other parameters are expressed as the mean ± SD. Since the primary objective of this study was to assess the advantage of SMTP‐7 over the control (saline treatment), statistical analyses were planned to examine differences between SMTP‐7 and saline groups at a one‐sided significance level of 5%. For this purpose, we used Wilcoxon rank sum test for the analysis of neurologic deficits and bleeding time, and Student's *t*‐test for the analysis of clot size, infarct size, edema, and blood parameters. We performed additional post‐hoc multiple comparison analyses. Details of the methods used are given where mentioned. In the correlation analyses, *P*‐values were obtained based on Pearson correlation coefficients (*r*) or Spearman correlation coefficients (*ρ*) at a two‐sided significance level of 5%. Statistical analyses were performed using SAS System for Windows (Release 9.2; SAS Institute, Inc., Cary, NC) or BellCurve for Excel (Social Survey Research Information Co., Ltd., Tokyo, Japan).

## 3 RESULTS

### 3.1 Dosing‐related death

Two prematurely died animals in the t‐PA group had massive hemorrhagic infarct (Figure S2) and were counted as a dosing‐related death. Embolized thrombi were cleared from the MCA in these animals, suggesting an occurrence of thrombolysis by the drug treatment (Figure S2). There were no animals that died with intracranial hemorrhage in the saline and SMTP‐7 groups.

### 3.2 Clot clearance

**Figure 1:**
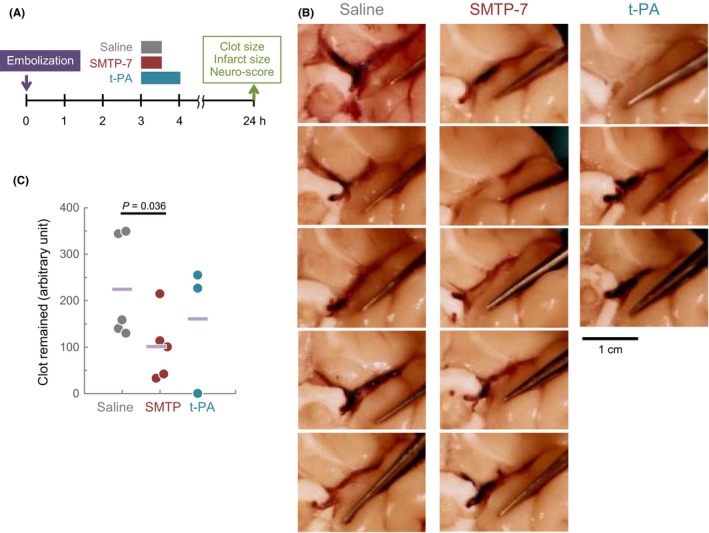
Thrombus remained in the MCA 24 hours after the embolization. (A) The experimental design. (B) Macroscopic observation of the external appearance of the origin of the MCA in individual animals. Bar, 1 cm. (C) Summary of the quantified data for clot remaining. Circles indicate individual values, and horizontal bars represent the mean. *P*‐value by unpaired Student's t‐test is shown. MCA, middle cerebral artery

Clot clearance was investigated by measuring the clots remained in the MCA 24 hours after the thrombus embolization. The amounts of the injected thrombus remaining were 224 ± 112 AU in the saline group, 109 ± 73 AU in the SMTP group, and 161 ± 140 AU in the survived animals in the t‐PA group (Figure 1). The difference between the saline and SMTP‐7 group was significant (*P* < 0.05).

### 3.3 Infarct and edema development

**Figure 2:**
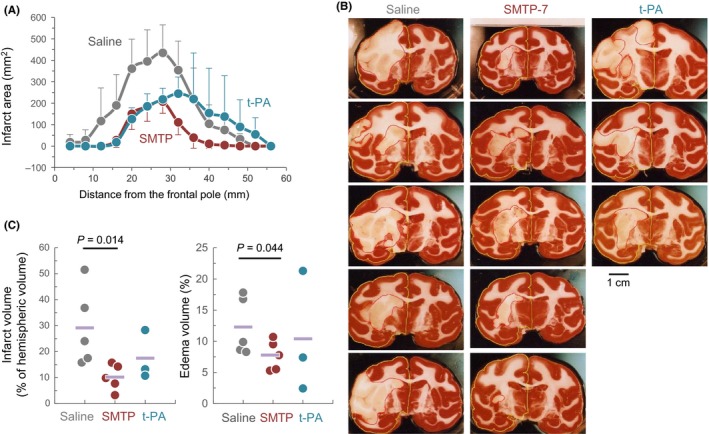
Sizes of cerebral infarct and edema 24 hours after the embolization. (A) Infarct area in each of the coronal brain sections, which were numbered from the frontal pole. (B) Images of the 8th TTC‐stained coronal sections (28–32 mm from the frontal pole) from each animal. Red and yellow lines outline infarct area and left hemispheric area, respectively. (C) The summary of the quantified data for infarct volume and edema volume. Circles indicate individual values, and horizontal bars represent the mean. *P*‐values by unpaired Student's *t*‐test are shown

The distribution of infarct area in each of the 14 brain slices obtained 24 hours after the embolization is summarized in Figure 2A. Infarct areas in the brain of the saline and SMTP‐7 groups peaked around 20‐36 mm from the frontal pole, whereas those in the t‐PA‐treated animals shifted to the posterior side (Figure 2A and S3). Photographs of the 8th TTC‐stained section (28‐32 mm from the frontal pole) in each animal are shown in Figure 2B. The infarct volume (% of total ipsilateral brain volume), calculated from all the TTC stained sections, were 29.1% to 13.4% in the saline group, 10.1% to 4.5% in the SMTP‐7 group, and 17.4% to 7.4% in the t‐PA group (Figure 2C). The difference between the saline and SMTP‐7 group was statistically significant (*P* < 0.05). Figure 2D shows the relative volume of edema, which were 12.3% to 4.1% in the saline group, 7.7% to 2.1% in the SMTP‐7 group, and 10.4% to 7.0% in the t‐PA group. The difference between the saline and SMTP‐7 group was significant (*P* < 0.05).

### 3.4 Neurologic deficits

**Figure 3:**
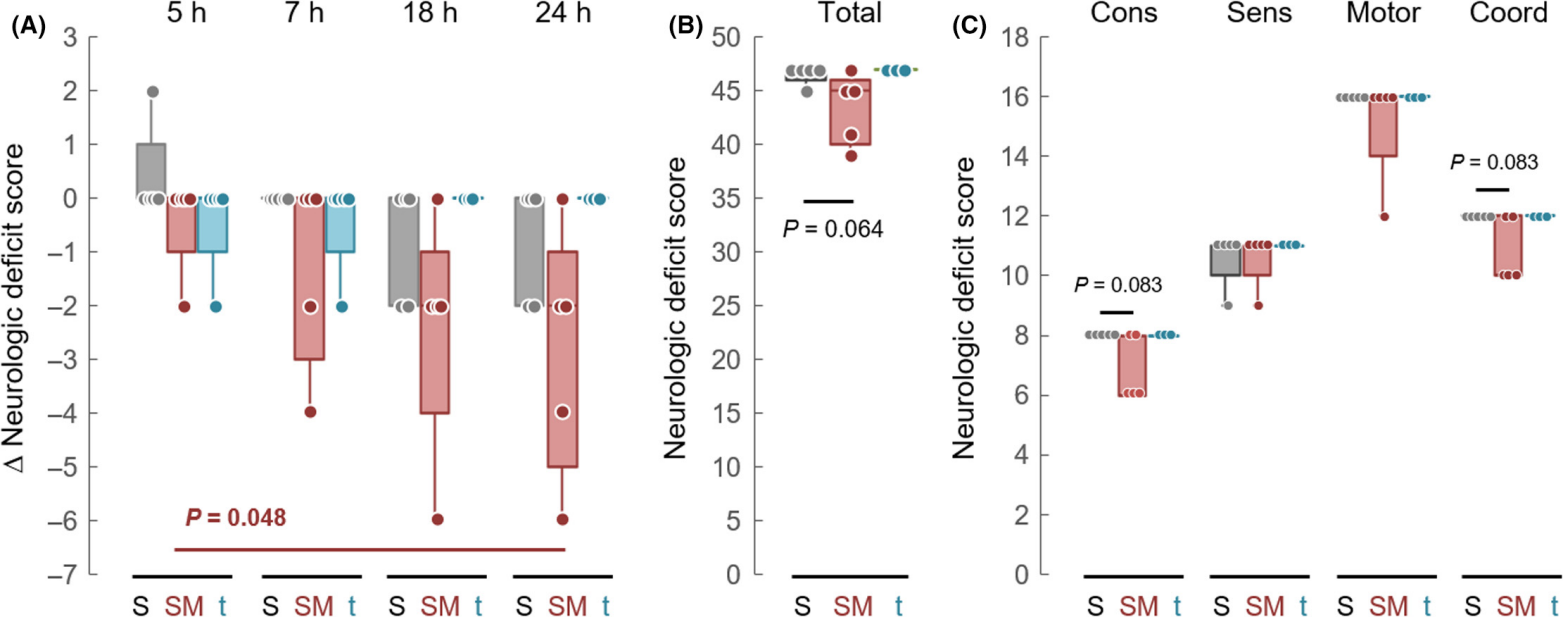
Neurologic deficit scores. (A) Change in the neurologic deficit scores after the drug treatment. Each value represents the difference from the neurologic deficit score observed at 3 hours after the embolization (just before the drug administration). *P*‐value for intragroup (SMTP‐7 group) variance by Friedman test is shown. No significant intragroup variance was found in the t‐PA or saline group. (B) Total of the neurologic deficit scores determined at 24 hours after the embolization. (C) Neurologic deficit scores for consciousness (Cons), the sensory system (Sens), the motor system (Motor), and skeletal muscle coordination (Coord) determined at 24 hours after the embolization. *P*‐values by Wilcoxon rank sum test are shown. Circles indicate individual values, and box and vertical bar represent IQR and the maximum/minimum, respectively. The median (horizontal line) is obscured by circles

Neurologic deficits were monitored before (3 hours after the embolization) and after the dosing (5, 7, 18, and 24 hours after the embolization). While neurologic deficit score in the saline and t‐PA groups remained constant during the observation period, the score tended to decline in the SMTP‐7 group (*P* = 0.048 for intragroup variance by Friedman test, and *P* = 0.072 by Scheffe pairwise multiple comparison of the difference between the 3 and 24‐hour time points) (Figure 3A). At the 24‐hour time point, the median total neurologic deficit score was 47 points (IQR: 47‐47) in the saline group, 45 points (IQR 41‐45) in the SMTP‐7 group, and 47 points (IQR 47‐47) in the t‐PA group (Figure 3B). The significance level in the difference between the saline and SMTP‐7 groups was *P* = 0.064 by Wilcoxon rank sum test. Among the subcategories of the neurologic deficits, differences between the saline and SMTP‐7 groups in both consciousness and skeletal muscle coordination were at *P* = 0.083 (Figure 3C).

### 3.5 Bleeding

We measured bleeding time just after the thrombus embolization and just before the termination of the drug infusion, since the drugs used could be categorized as thrombolytics. We found no significant intragroup dosing‐related change in the bleeding time in each of the three groups (by Friedman test) as well as intergroup differences in the post‐dosing values (by Wilcoxon rank sum test) (Figure S4).

### 3.6 Blood parameters

**Figure 4:**
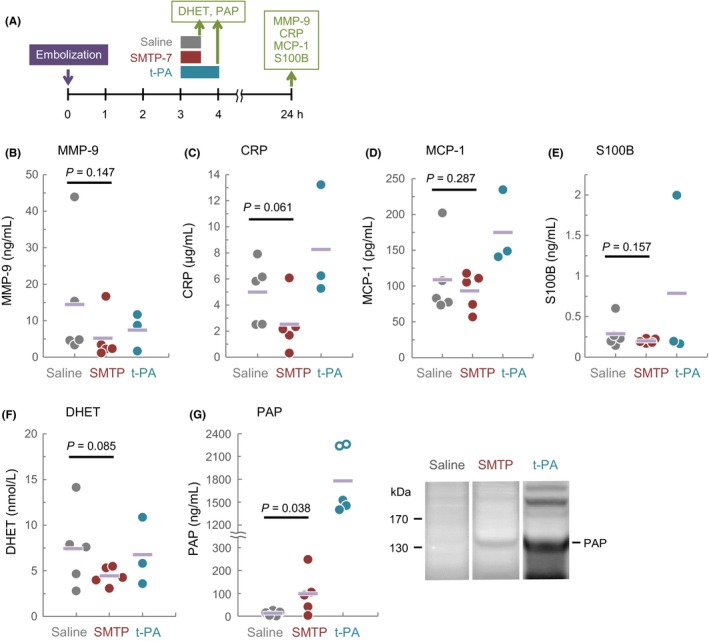
Blood parameters. (A) The study design. (B‐G) Plasma levels of MMP‐9, CRP, MCP‐1, S100B, DHET, and PAP. Inset of panel G shows representative results of the zymography, in which PAP appeared as a ~135 kDa band. Additional bands were observed in the lane for plasma from a t‐PA‐treated animal, suggesting an extreme impact on the hemostatic system. Circles indicate individual values, and horizontal bars represent the mean. Open circles in panel G (for the t‐PA group) represent data for prematurely died animals. *P*‐values by unpaired Student's *t*‐test are shown. *P*‐value based on Pearson correlation coefficients (*r*) is shown. CRP, C‐reactive protein; DHET, dihydroxyeicosatrienoic acid

To seek a biomarker that predicts efficacy of SMTP‐7, we tested the following substances for their plasma levels after the drug treatment: PAP, DHET, MMP‐9, CRP, MCP‐1, and S100B (Figure 4A). PAP is formed from plasmin and its physiological inhibitor α_2_‐antiplasmin, representing plasminogen activation.^30^ DHET is a product of sEH‐mediated hydrolysis of the anti‐inflammatory fatty acid epoxide epoxyeicosatrienoic acid, representing sEH inhibition.^31^ MMP‐9, a zinc‐binding protease that degrades components of the basal lamina around blood vessels, can be a marker of ischemic stroke development as well as its hemorrhagic transformation.^32^ The acute‐phase reactant CRP can be a marker of ischemic stroke development and a predictor of stroke prognosis.^33^ A high level of the chemokine MCP‐1 is found in ischemic stroke patients, and it can mirror the development of inflammatory response in the brain.^34^ The level of S100B can predict a course of infarction in ischemic stroke patients.^35^

The average levels of MMP‐9, CRP, MCP‐1, S100B, and DHET in the SMTP‐7 group were 36%, 50%, 86%, 69%, and 60% of the respective levels in the saline group, and the respective levels in the t‐PA group were 51%, 165%, 161%, 273%, and 91% of saline control (Figure 4B–F). Regarding the differences in CRP and DHET levels, differences between the saline and SMTP‐7 groups were at *P* = 0.061 and 0.085, respectively. The level of PAP in the SMTP‐7 group (98.1 ± 93.7 ng/mL) was significantly higher than that in the saline group (12.4 ± 10.5 ng/mL; *P* < 0.05) (Figure 4G).

### 3.7 Correlations among stroke pathologies and blood parameters

**Figure 5:**
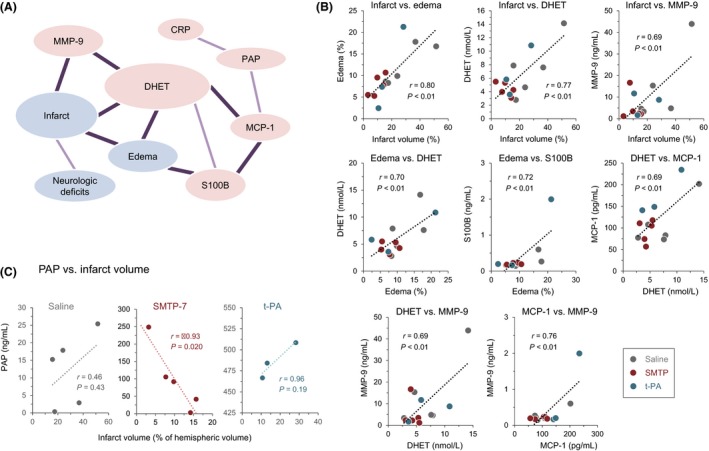
Correlations among the stroke pathologies and blood biochemical parameters. (A) Schematic summary of the results, where significant correlations are shown as bold lines (*P* < 0.01) or plane lines (*P* < 0.05). Clot, clot remaining in the MCA; Infarct, infarct volume; Edema, edema volume; and Neuro, neurologic deficit score. (B) Graphical representation of the significant correlations. (C) Correlation between infarct volume and the level of PAP. Note the difference in the scale (both ordinate and abscissa) among the three graphs. *r*, Pearson correlation coefficients. MCA, middle cerebral artery

To characterize relationships among the stroke pathologies and blood biochemical parameters, we analyzed possible correlations using the data obtained from individual animals of all of the treatment groups (Table S2). The results demonstrated a network covering infarct, edema, DHET, MMP‐9, S100B, and MCP‐1 (Figure 5A,B). The correlations between MCP‐1 and PAP, PAP, and CRP, and infarct and neurologic deficits extended this network. These results suggest a relationship between the developments of stroke pathology and inflammation in this model. When analyzed for each treatment group (Table S2), a significant inverse correlation between PAP level and infarct volume (*r* = 0.93; *P* < 0.05) was found in the SMTP‐7 group (Figure 5C). The PAP level in the t‐PA group was profoundly high (1459 ± 63.2 ng/mL for the three survived animals) (Figure 4G), whereas neither significant correlation was found between PAP and infarct volume in the t‐PA group nor saline group (Figure 5C).

## 4 DISCUSSION

We investigated efficacy of SMTP‐7 in a monkey model of embolic stroke, which was severe in that a large thrombus (10 cm in length) was introduced to occlude the MCA. Moreover, the timing of drug treatment was set at 3 hours following embolization, which was too late to achieve good outcome by the treatment with t‐PA. Indeed, two animals of five tested in the t‐PA group prematurely died in this experiment, and none of the pathological parameters improved in the remaining three survived animals as compared with the saline‐treated animals in post‐hoc analyses, setting a one‐sided significance level of 5%. The animals that prematurely died developed hemorrhagic infarction, while the injected thrombus was cleared (Figure S2). A negative correlation was found between clot size and infarct volume in the survived animals in the t‐PA group (Table S2). These findings suggest an involvement of reperfusion injury that leads to poor outcome in the t‐PA treated animals.

SMTP‐7 was effective in alleviating pathological conditions associated with the thrombus embolization. The SMTP‐7‐treated animals had significantly reduced sizes of infarction, edema, and clot remaining in the MCA as compared to those of the saline‐treated animals. There may be some differences in the actions of SMTP‐7 and t‐PA that lead to the different outcomes. One possibility is the difference in the thrombolytic mechanism that may affect severity of ischemia‐reperfusion injury. t‐PA treatments cause reductions in plasma levels of the hemostatic factors such as α_2_‐antiplasmin, plasminogen, and fibrinogen.^27^, ^36^, ^37^ These declines can be the consequences of systemic activation of plasminogen. The action of SMTP‐7 is to enhance endogenous fibrinolytic system; it is not a direct plasminogen activator but a modulator that relaxes plasminogen conformation.^8^, ^9^ This could lead to plasminogen activation only where, when, and to the extent endogenous plasminogen activators are supplied.^6^ Indeed, SMTP‐7 does not reduce the levels of α_2_‐antiplasmin and fibrinogen in normal monkeys.^17^ In consistent with this notion, we observed a moderate elevation of PAP level in the SMTP‐7 group, whereas the elevation of PAP in the t‐PA group was strikingly high (Figure 4G). As for ischemia‐reperfusion injury in the brain, a gradual reperfusion can be safer than a rapid flow restoration.^38^ These findings suggest the benefit of physiological thrombolysis compared to inordinate thrombolysis arising from t‐PA treatment.

Another possibility is the involvement of the anti‐inflammatory and anti‐oxidative actions of SMTP‐7.^11^, ^19^, ^39^, ^40^ Inflammatory and oxidative reactions play crucial roles in the development of ischemia‐reperfusion injury.^41^ Indeed, there are several correlation networks that tie stroke pathologies with inflammatory responses in the embolic stroke model used in this study (Figure 5A), suggesting a role of inflammation in stroke development in this model. Although t‐PA is proinflammatory to cerebral tissues,^42^ SMTP‐7 suppresses inflammatory and oxidative responses in stroke models.^10^, ^13^, ^16^, ^19^, ^20^ As shown in Figure 4, our results are in line with this idea.

In conclusion, our results demonstrate an efficacy of SMTP‐7 in a severe embolic stroke model where recanalization therapy may cause ischemia‐reperfusion injury that impairs outcome. There are, however, some issues that require considerations: (1) the conclusion have been drawn from an experiment with a limited number of animals for ethical reasons in the use of primates; (2) etiology and pathology of stroke in humans are heterogeneous, whereas uniform animal model is used in this study; (3) while most of ischemic stroke patients are the elderly, this study was conducted using relatively young animals; and (4) female animals were not used whereas in humans females have a larger stroke burden than males. Using an SMTP compound, a phase 2 clinical trial enrolling aged male and female ischemic stroke patients is currently under investigation (JapicCTI‐183842, at the Japan Pharmaceutical Information Center Clinical Trials Information). This study may reveal therapeutic potential of the SMTP compound. Regarding the issue (1), results of neurologic measurements are trending to show an SMTP‐7 efficacy, and increasing sample size may be required for increasing the power of detection in future primate studies of this kind. A similar notion may be applied to other trending data such as DHET, MMP‐9, and S100B levels as well as the correlation analyses, emphasizing again the importance of securing the number of animals.

## AUTHORS CONTRIBUTION

Participated in research design: Suzuki, Nishimura, Yoshikawa, and Hasumi. Conducted experiments: Yoshikawa and Kunikiyo. Contributed new reagents or analytic tools: Nishimura, Hasegawa, and Hasumi. Performed data analysis: Suzuki, Nishimura, Yoshikawa, Kunikiyo, Hasegawa, and Hasumi. Wrote or contributed to the writing of the manuscript: Suzuki, Nishimura, and Hasumi.

## DISCLOSURE

The authors declare no conflict of interest.
