# Supplementary material for: Efficacy of SMTP‐7, a small‐molecule anti‐inflammatory thrombolytic, in embolic stroke in monkeys
Source: Pharmacol Res Perspect. 2018 Dec 5;6(6):e00448. doi: 10.1002/prp2.448 (PMC6282002; doi:10.1002/prp2.448)
Supplement: Supplementary file 1 [file PRP2-6-e00448-s001.pdf]

Supporting information for

Efficacy of SMTP-7, a small-molecule anti-inflammatory thrombolytic, in embolic stroke in monkeys

Eriko Suzuki[1,*], Naoko Nishimura[2,*], Tetsuya Yoshikawa[3], Yudai Kunikiyo[1], Keiko Hasegawa[2], and Keiji Hasumi[1,2]

[1]Department of Applied Biological Science, Tokyo Noko University, Tokyo, Japan; [2]Division of Research and Development, TMS Co., Ltd., Tokyo, Japan; and [3]Shin Nippon Biomedical Laboratories, Ltd., Kagoshima, Japan

*These authors contributed equally to this work.

Correspondence: Keiji Hasumi, Department of Applied Biological Science, Tokyo Noko University, 3-5-8 Saiwaicho, Fuchu, Tokyo 183-8509, Japan; and TMS Co., Ltd., 1-23-3-501 Miyamachi, Fuchu, Tokyo 183-0005, Japan

Tel.: +81 42 367 5710; Fax: +81 42 367 5708

E-mail: hasumi@cc.tuat.ac.jp

Supplementary Table 1. Neurologic deficit scoring

Supplementary Table 2. Correlations among the stroke pathologies and blood biochemical parameters

Supplementary Figure 1. Macroscopic appearance of the origin of the MCA in animals excluded for the criterion 2.

Supplementary Figure 2. Macroscopic appearance of the brains from the animals taken as dosing-related death

Supplementary Figure 3. Images of the TTC-stained coronal brain sections from a representative animal in each group

Supplementary Figure 4. Bleeding time before and after dosing

Supplementary Table 1. Neurologic deficit scoring

The evaluation parameters comprised 4 categories; consciousness, sensory system, motor system, and skeletal muscle coordination as shown below. The sum of the scores in all categories was calculated as the total neurologic deficit score.

**Consciousness (range 0–28)**: observed at the cage side and evaluated in accordance with the criteria stated below.	
0	Normal consistently alert
4	Conscious and aggressive
6	Conscious and escape
8	Conscious but clouded and accepting
10	Drowsiness, aroused with stimulation
16	Lethargia, eyes open by strong stimulation
20	Stuporous, aroused with persistent stimulation
24	Light coma, reflex movement only
28	Deep coma, no movement
**Sensory system (range 0–22)**: animals were restrained by hand and the evaluation was performed using forceps to touch or pinch the ipsilateral (left side at which the clot was infused) and contralateral (right) sides to brain embolism, in accordance with the criteria stated below.	
Facial sensation (ipsilateral/contralateral)	
0/0	Reacts consistently to touch in any of face
3/3	Absent, does not react to touch in any area of face
Pinna reflex (ipsilateral/contralateral)	
0/0	Twitches ear in response to outer/inner hairs
3/3	Absent, does not move ear in response to touch
Pain reflex (lower limb, ipsilateral/contralateral)	
0/0	Strong, quick, complete withdrawal from toe pinch
3/3	Weak, slow, incomplete, or inconsistent withdrawal from toe pinch
5/5	Absent, no withdrawal from toe pinch
**Motor system (range 0–32)**: animals were restricted by hand and the evaluation was performed by touching the ipsilateral (left) and contralateral (right) sides to brain embolism, in accordance with the criteria stated below.	
Hand (motor power/movement, ipsilateral/contralateral)	
0/0	Normal
2/2	Reduced strength/skilled
4/4	Paralysis/useless
Leg (motor power/movement, ipsilateral/contralateral)	
0/0	Normal
2/2	Raises with flexion of knee/against gravity
4/4	Can move, but not against gravity/impossible
6/6	Paralysis/useless
Upper limb tone (ipsilateral/contralateral)	
0/0	Normal
3/3	Overtly spastic or flaccid
Lower limb tone (ipsilateral/contralateral)	
0/0	Normal
3/3	Overtly spastic or flaccid
**Skeletal muscle coordination (range 0–18)**: observed at cage side and evaluated in accordance with the criteria stated below.	
0	Normal, walks normally
4	Minimal ataxia, walks with some impairment of gait
6	Ataxia, but able to climb the wire net
10	Stands spontaneously, falls with a few steps
12	Sits, just able to circle
16	Posed lateral or dorsal recumbency
18	No movement

Supplementary Table 2. Correlations among the stroke pathologies and blood biochemical parameters

The results of the analyses of the data for all of the three treatment groups as well as each of the treatment groups are shown. Numerical data are correlation constants that represent Pearson correlation coefficients (r) or Spearman rank correlation coefficients (ρ) (for correlation concerning neurologic deficit score). Values in blue characters, $P < 0.05$; and values in red characters, $P < 0.01$. *Clot*, clot remaining in the MCA; *Infarct*, infarct volume; *Edema*, edema volume; and *Neuro*, neurologic deficit score.

All groups

	Infarct	Edema	Neuro	PAP	DHET	CRP	MCP-1	MMP-9	S100B
Clot	0.23	−0.26	0.43	−0.03	0.09	0.37	0.10	0.42	−0.33
Infarct		0.80	0.46	−0.12	0.77	−0.06	0.43	0.69	0.39
Edema			0.26	0.02	0.70	−0.21	0.46	0.30	0.72
Neuro				−0.15	0.04	0.52	0.45	0.16	0.41
PAP					0.12	0.56	0.64	−0.11	0.50
DHET						−0.05	0.69	0.69	0.59
CRP							0.21	−0.05	0.00
MCP-1								0.48	0.76
MMP-9									0.18

Saline group

	Infarct	Edema	Neuro	PAP	DHET	CRP	MCP-1	MMP-9	S100B
Clot	0.29	−0.01	0.35	0.07	0.18	−0.27	0.53	0.50	0.63
Infarct		0.90	0.71	0.46	0.83	−0.27	0.78	0.82	0.91
Edema			0.35	0.16	0.68	−0.14	0.45	0.49	0.67
Neuro				0.00	−0.35	0.35	0.00	0.35	0.71
PAP					0.70	−0.44	0.81	0.81	0.53
DHET						−0.72	0.81	0.81	0.82
CRP							−0.47	−0.41	−0.45
MCP-1								1.00	0.91
MMP-9									0.92

SMTP-7 group

	Infarct	Edema	Neuro	PAP	DHET	CRP	MCP-1	MMP-9	S100B
Clot	−0.77	−0.83	0.36	0.77	0.22	0.69	0.51	−0.01	−0.53
Infarct		0.77	0.31	−0.93	−0.65	−0.75	−0.49	−0.21	0.45
Edema			0.05	−0.59	−0.02	−0.35	−0.37	−0.50	0.57
Neuro				−0.46	−0.41	−0.15	0.31	−0.56	−0.05
PAP					0.77	0.94	0.40	−0.03	−0.50
DHET						0.80	0.27	−0.26	-0.09
CRP							0.30	−0.30	−0.45
MCP-1								−0.41	0.43
MMP-9									−0.53

t-PA group

	Infarct	Edema	Neuro	PAP	DHET	CRP	MCP-1	MMP-9	S100B
Clot	−1.00	−0.99	NA	−0.95	−0.92	0.67	−0.98	−0.13	−0.99
Infarct		0.99	NA	0.96	0.91	−0.69	0.98	0.10	0.99
Edema			NA	0.98	0.85	−0.78	0.95	−0.02	0.96
Neuro				NA	NA	NA	NA	NA	NA
PAP					0.74	−0.88	0.87	−0.20	0.90
DHET						−0.32	0.97	0.51	0.96
CRP							−0.53	0.65	−0.58
MCP-1								0.31	1.00
MMP-9									0.25

Value in blue, $P < 0.05$; value in red, $P < 0.01$

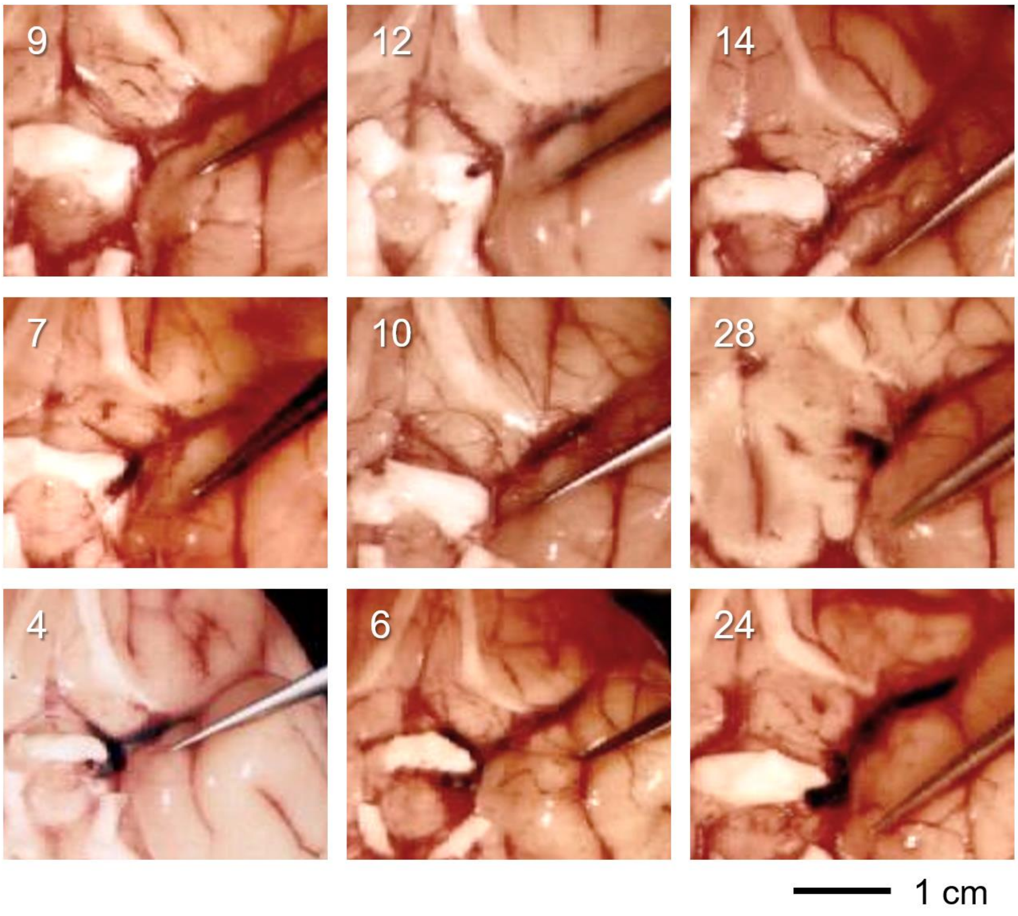

Supplementary Figure 1. Macroscopic appearance of the origin of the MCA in animals excluded for the criterion 2.

Photographs of the origin of the MCA in animals excluded for the criterion 2 (judged unsuitable to continue the study during the observation period) are shown. The number corresponds to the animal number shown in Supplementary Table 2. Bar, 1 cm.

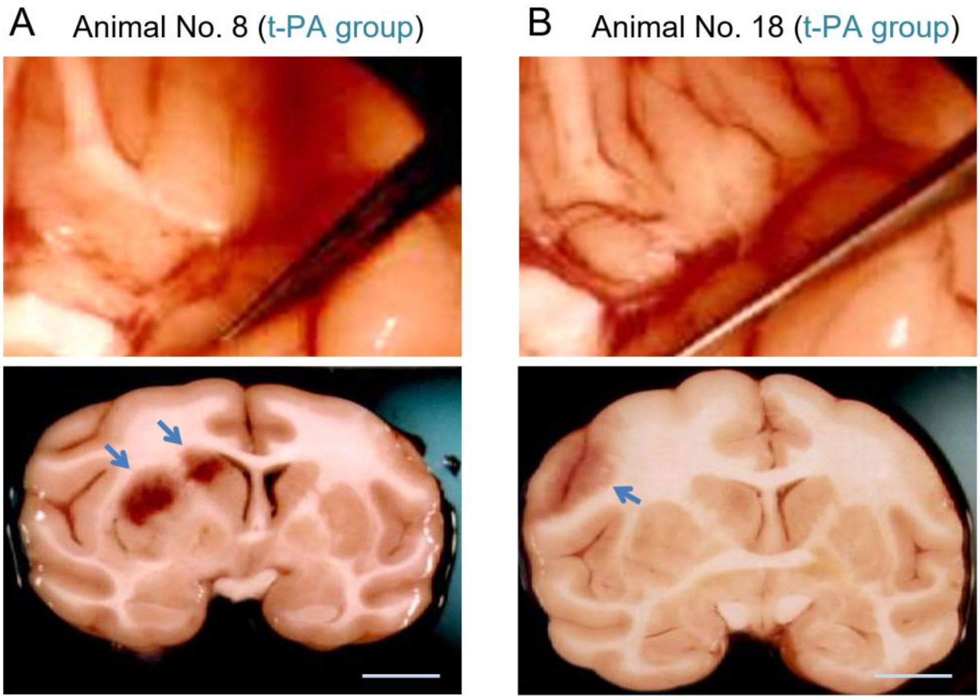

Supplementary Figure 2. Macroscopic appearance of the brains from the animals taken as dosing-related death

Photographs of the origin of the MCA (*upper*) and the 7th section (24–28 mm from the frontal pole) of the brain slices (*lower*) from the animals taken as "dosing-related death" are shown. (A) Animal No. 8 in the t-PA group, which died 7–18 h after the embolization. (B) Animal No. 18 in the t-PA group, which died 7–18 h after the embolization. Prominent hemorrhagic signs were observed in the sections of both animals, while there was no apparent clot remaining in the MCA region. Arrows indicate hemorrhagic infarct area. Bar, 1 cm.

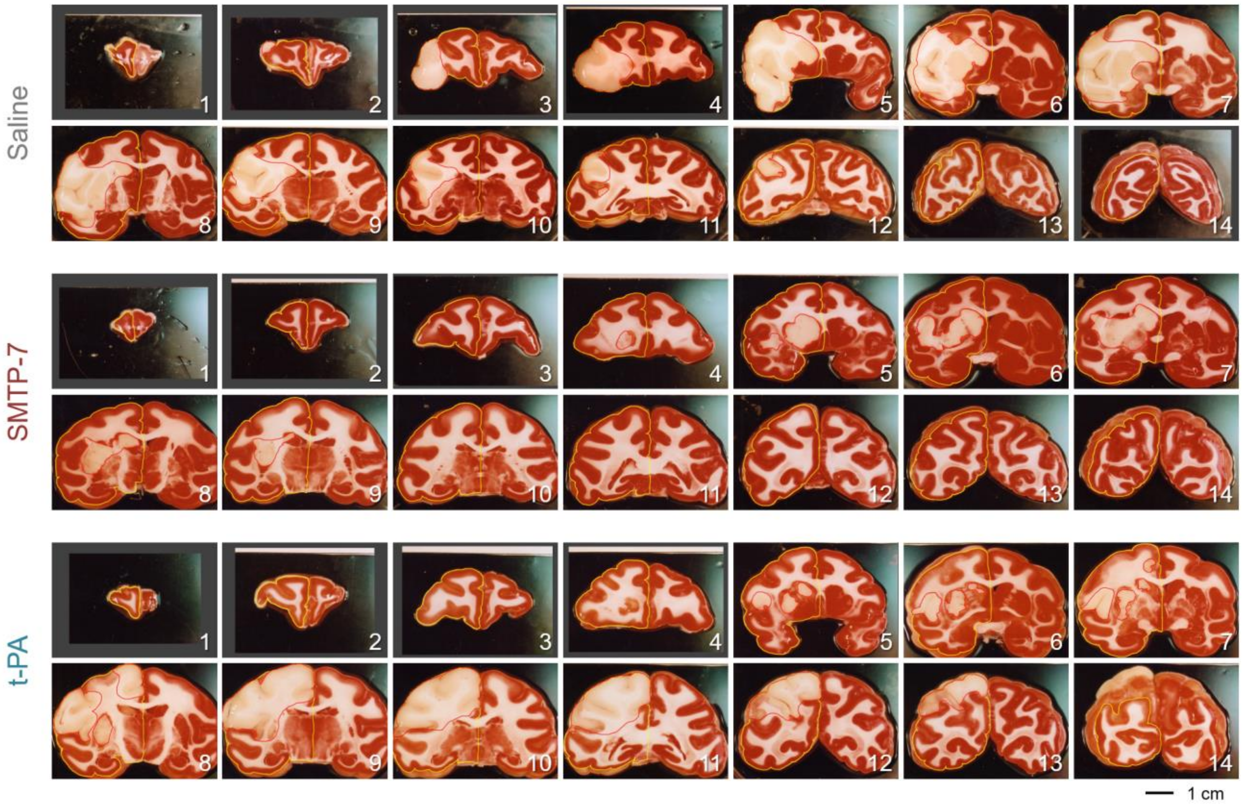

Supplementary Figure 3. Images of the TTC-stained coronal brain sections from a representative animal in each group

All of the TTC-stained 14 coronal brain sections from representative animals in each of the saline, SMTP-7, and t-PA groups are shown. Infarct areas in the brain of the saline and SMTP-7 groups peaked around 20–36 mm from the frontal pole, whereas those in the t-PA-treated animals shifted to the posterior side. Number at the bottom right represents the brain slice number. Red and yellow lines outline infarct area and left hemispheric area, respectively. Bar, 1 cm.

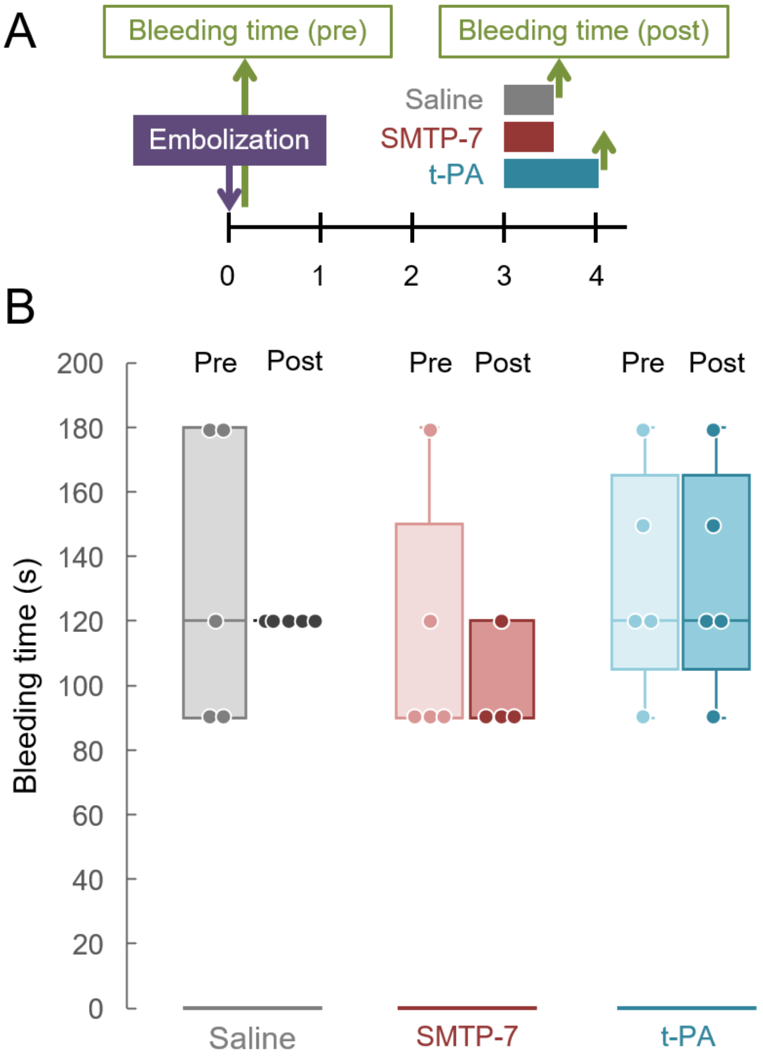

Supplementary Figure 4. Bleeding time before and after dosing

Male cynomolgus monkeys were subjected to the bleeding test before (just after the thrombus embolization) (*Pre*) and after the drug administration (*Post*). (A) Study outline. (B) The results of the bleeding test. Circles indicate individual values, and box, vertical bar, and horizontal bar represent IQR, the maximum/minimum, and the median, respectively. No statistically significant difference was found between the saline and SMTP-7 groups (Wilcoxon rank sum test) or among any of the three groups (Kruskal-Wallis test).
